# Editorial: Functions of Non-Coding RNA in Innate Immunity

**DOI:** 10.3389/fimmu.2015.00622

**Published:** 2015-12-14

**Authors:** Susan Carpenter

**Affiliations:** ^1^Department of Molecular, Cell and Developmental Biology, University of California, Santa Cruz, Santa Cruz, CA, USA

**Keywords:** innate immunity, long non-coding RNAs, miRNAs, inflammation, extracellular RNA

Our understanding of the functions that non-coding RNAs play in shaping the immune response is still in its infancy. The breakthroughs in deep sequencing technology have provided us with an unprecedented view of the human genome. The deeper we sequence the more non-coding genes we identify, while the number of protein coding genes remains constant. GENCODE represents the gene set of the ENCODE project and there are currently 15,931 long non-coding RNA (lncRNA) genes, 9,882 small non-coding RNA genes, and 14,477 Pseudogenes cataloged in GENCODE version 23^1^. The contribution of each of these genes to biological processes still remains to be determined. In this research topic, we explore recent data surrounding the functions for microRNA (miRNA) as well as lncRNA within Innate Immunity.

Innate immune responses to infection involve the production of pro-inflammatory cytokines such as IL-6 and TNFα in addition to the Type I Interferons (IFNs) that play critical roles in anti-viral immunity. In recent years, there have been huge strides made in understanding the contributions of small RNAs such as miRNA to the Innate Immune processes. More recently, our attention has also been drawn to the growing catalog of lncRNAs. miRNAs are ~22 nt in length are function through post-transcriptional regulation of protein coding genes through regulating translation and RNA stability. LncRNA are transcripts greater than 200 nt in length that do not encode for protein. Both miRNAs and lncRNAs are RNA pol II transcripts, that are capped and many are polyadenylated; however, there is also evidence that both lncRNA and miRNA can be transcribed by RNA polymerase III (1, 2). The vast functions for miRNA and lncRNA within the innate immune responses are thoroughly reviewed in this research topic by Foster et al., Stachurska et al., and Imamura and Akimitsu (3–5).

## Non-Coding RNA and Interferon

It is critical that innate immune signaling remains transient as any perturbations to these complex pathways can have devastating consequences for the host. For this reason, there are many positive and negative feedback mechanisms in place to keep the pathways in check. miRNAs can act as fine tuners of immune signaling. Type I IFNs are critical for protection against viral infection and more than 30 miRNAs have been shown to be differentially regulated by IFN stimulation with many of these targeting IFN-β acting as negative feedback regulators (3, 6). There are three original research articles within this research topic that center on IFN inducible lncRNAs and like miRNAs they appear to form critical functions as key regulators of the anti-viral immune pathways (7–9). Interestingly, two independent research articles identify the same IFNα inducible lncRNA BST2 IFN-stimulated positive regulator (BISPR) (8, 9). A bi-directional promoter of the protein-coding gene BST2 transcribes BISPR that then acts as a positive regulator for BST2 expression. siRNA-mediated knockdown of BISPR had a dramatic impact on BST2 protein expression levels. The original articles in this series identify a large number of lncRNAs whose functions within anti-viral immunity remain to be determined.

Throughout this research topic, we return to the idea of regulatory loops, which includes miR21, which is capable of negatively regulating the pathway that is responsible for its induction (10). miR21 serves many functions within the innate immune response and these are reviewed in depth in this research topic (10). miRNAs and lncRNAs form their own complex regulatory loops with many lncRNAs being targets by miRNAs and vice versa. Circular RNA is a specialized type of lncRNA that can act as miRNA sponges increasing the complexity of these regulatory loops further (11). No functions for circRNA in innate immune responses have been identified to date.

## Non-Coding RNA in Disease and Development

A large number of non-coding RNAs are dysregulated in complex inflammatory diseases. The exact extent to which differentially regulated miRNA and lncRNA contribute to disease pathologies are still under intense investigation and reviewed here by Stachurska et al. (4). miR146a is a key regulator of the inflammatory response. Reduced levels of miR146 are associated with Systemic Lupus Erthyromatosis. The miR146 knockout mice display an autoimmune phenotype as well as developing tumors with age. The extended functions of miR146 in innate immunity are reviewed by Saba et al. (12).

miRNAs have been shown to act as key regulators of innate immune cell development. MiR-223 is involved in granulocyte production, the miR125 family, miR142, miR155, mir342, mir338, and miR145 all play roles in macrophage differentiation (4). To date, only two lncRNAs have been shown to be involved in innate immune cell differentiation. Lnc-DC was identified by Wang et al. in human conventional dendritic cells. Knockdown of lnc-DC results in a failure of monocytes to differentiate into conventional dendritic cells as well as affecting the ability of the cells to activate T cells (13). HOTAIRM1 can regulate retinoic acid-mediated granulocytic differentiation (14). More recently, lnc-MC has been shown to be required for macrophage differentiation (15). It acts as a competitive endogenous RNA sequestering miR-199a-5p from its protein target activin A receptor type 1B that is a critical regulator of monocyte/macrophage differentiation.

## Extracellular RNA

The study of extracellular RNA has intensified over recent years. Much interest is focused on trying to understand how these exRNAs can communicate and travel between cells, the impact they have as well as their possible use as easily accessible biomarkers for disease. The major species of RNA found within extracellular vesicles (EVs) are small RNA including miRNA. Recent evidence suggest that miR21 can be released from tumor cells within EVs and where it can act as a ligand for TLR7 and TLR8 in mice (16). A number of mRNA as well as lncRNA have also been identified within EVs. This topic is covered in depth by Van der Grein et al. (16). Intriguingly, there appears to be cell type specify in terms of the RNAs exported into EVs; however, the full extent of their impact on neighboring as well as far away cells has not been fully examined *in vivo*. Like all aspects of the immune response, this process can be hijacked by microbial pathogens for their own survival. Many viruses and bacteria have been shown to package RNAs into EVs, which are released into uninfected cells where they can contribute to immune evasion. Further work needed on extracellular RNAs how they impact immune signaling; can their presence be harnessed therapeutically? Can we design methods to easily detect these non-coding RNAs as diagnostic markers and could they represent targets for therapeutic intervention?

## Conclusion

Unlike the miRNA field our understanding of the molecular mechanisms employed by lncRNA are at a very early stage. LncRNAs can originate from intergenic regions between two protein coding genes, from divergent bi-directional promoters, which is the focus of a number of papers covered in this research topic (8, 9). LncRNA can overlap protein-coding genes and even arise from transcription of enhancer regions (eRNAs) (17). LncRNA can positively and negatively influence expression patterns of other genes both *in cis* and *in trans* through binding chromatin-modifying complexes (18). As the number of lncRNAs continues to rise so does the complexity of their functions. It is an exciting time for the non-coding RNA field and this special topic highlights many interesting aspects of non-coding RNA function within the immune system. What is clear that is more work is needed to understand the full extent to which these genes contribute to key biological processes as well as disease states.

## Conflict of Interest Statement

The author declares that the research was conducted in the absence of any commercial or financial relationships that could be construed as a potential conflict of interest.

## Footnotes

^1^http://www.gencodegenes.org/stats/current.html
